# NUSAP1 promotes pancreatic ductal adenocarcinoma progression by drives the epithelial-mesenchymal transition and reduces AMPK phosphorylation

**DOI:** 10.1186/s12885-024-11842-5

**Published:** 2024-01-16

**Authors:** Yuan Liu, Rong Tang, Qing-Cai Meng, Si Shi, Jin Xu, Xian-Jun Yu, Bo Zhang, Wei Wang

**Affiliations:** 1 Department of Endoscopy, Fudan University Shanghai Cancer Center, Shanghai, China; 2 Department of Oncology, Shanghai Medical College, Fudan University, Shanghai, China; 3 Shanghai Pancreatic Cancer Institute, No.270 Dong’An Road, Shanghai, 200032 China; 4 Department of Pancreatic Surgery, Fudan University Shanghai Cancer Center, Shanghai, China

**Keywords:** Pancreatic ductal adenocarcinoma, Differentially expressed genes, Functional enrichment analysis, Protein‒protein interaction, Survival analysis

## Abstract

**Background:**

Pancreatic ductal adenocarcinoma (PDAC) has a poor prognosis, and its molecular mechanisms are unclear. Nucleolar and spindle-associated protein 1 (NUSAP1), an indispensable mitotic regulator, has been reported to be involved in the development of several types of tumors. The biological function and molecular mechanism of NUSAP1 in PDAC remain controversial. This study explored the effects and mechanism of NUSAP1 in PDAC.

**Methods:**

Differentially expressed genes (DEGs) were screened. A protein‒protein interaction (PPI) network was constructed to identify hub genes. Experimental studies and tissue microarray (TMA) analysis were performed to investigate the effects of NUSAP1 in PDAC and explore its mechanism.

**Results:**

Network analysis revealed that NUSAP1 is an essential hub gene in the PDAC transcriptome. Genome heterogeneity analysis revealed that NUSAP1 is related to tumor mutation burden (TMB), loss of heterozygosity (LOH) and homologous recombination deficiency (HRD) in PDAC. NUSAP1 is correlated with the levels of infiltrating immune cells, such as B cells and CD8 T cells. High NUSAP1 expression was found in PDAC tissues and was associated with a poor patient prognosis. NUSAP1 promoted cancer cell proliferation, migration and invasion, drives the epithelial-mesenchymal transition and reduces AMPK phosphorylation.

**Conclusions:**

NUSAP1 is an essential hub gene that promotes PDAC progression and leads to a dismal prognosis by drives the epithelial-mesenchymal transition and reduces AMPK phosphorylation.

**Supplementary Information:**

The online version contains supplementary material available at 10.1186/s12885-024-11842-5.

## Introduction

Pancreatic ductal adenocarcinoma (PDAC) is the most common cause of death from digestive system malignancies. It has been reported that the mortality rate of PDAC reached 93.95% in 2020 [1]. The median 5-year survival for stage 4 PDAC is 9% [2]. PDAC has a poor prognosis partly due to its rapid progression and the lack of diagnostic and therapeutic targets [3]. Although a lot of work has been done to reveal the pathogenesis of PDAC, there are still many unclear areas. Ectopically expressed genes involved in the cell cycle, development, cell differentiation/proliferation, and energy metabolism are among the factors involved in PDAC pathogenesis [4]. The key to developing more effective diagnostic and therapeutic strategies is to identify novel genes or specific targets of PDAC and clarify their roles [5].

Recently, gene chips and gene profiles have been widely used to screen differentially expressed genes (DEGs), and using these data, we can obtain new insights into the mechanism and treatment of PDAC [6]. Many bioinformatic studies of PDAC have been proven to be effective and reliable [7]. Because of the complex tumor heterogeneity and complicated molecular regulatory mechanism of PDAC, current studies may be insufficient or inconsistent. An integrated bioinformatics analysis could not only assist with exploring the biomarkers and the mechanisms underlying the tumorigenesis and progression of cancer but could also help to find novel and potential treatment options for the disease [8].

Nucleolar and spindle-associated protein 1 (NUSAP1) has recently been recognized as a cell cycle-regulating protein that binds microtubules and controls mitotic progression, spindle formation, and stability. The structural integrity of the spindle ensures the equal division of chromosomes, which can be a prerequisite for cell division. Abnormal spindle structure can result in incorrect chromosome separation (also known as chromosomal instability), which will lead to tumorigenesis [9]. Increased expression of NUSAP1 has been reported in prostate cancer, breast cancer, oral squamous cell carcinoma, and cervical cancer and is closely associated with tumor development and a poor prognosis [10]. However, studies on NUSAP1 in PDAC are rare, and its role in the mechanism of occurrence and development of PDAC remains unclear.

In this study, three original gene expression profiles (GSE15471, GSE16515, and GSE71989) were downloaded from the Gene Expression Omnibus (GEO) database. DEGs were screened, protein-protein interaction (PPI) network was constructed and hub genes were identified. Then, we experimentally identified the function of NUSAP1 in the carcinogenesis of PDAC. Our results will help to develop novel therapeutic strategies to improve clinical outcomes and provide new insights into PDAC biology.

## Materials and methods

### Data acquisition from the Gene expression Omnibus (GEO) and the Cancer Genome Atlas (TCGA)

In this study, we collected three microarray datasets (GSE15471, GSE16515 and GSE71989) from the GEO database (https://www.ncbi.nlm.nih.gov/geo/), which is a public repository of high-throughput gene expression genomics datasets. The inclusion criteria for the above gene expression profiles were set as follows: (1) the dataset included tissue samples obtained from human PDAC tissues and normal tissues; (2) the number of samples in each dataset was more than 8; (3) the platform was GPL570 (Affymetrix Human Genome U133 Plus 2.0 Array). The miRNA transcriptome data for 183 pancreatic-related samples (179 tumor tissues and 4 normal tissues) and the corresponding clinical information were also downloaded from the TCGA database (https://portal.gdc.cancer.gov/) on June 1, 2021.

### Identification of differentially expressed genes (DEGs)

GEO2R (https://www.ncbi.nlm.nih.gov/geo/geo2r/) is an online analysis tool based on the R programming language, and we used it to analyze DEGs that can distinguish between PDAC tissue samples and nontumor tissue samples. Volcano plots were drawn [11]. An adjusted *p* value of *<* 0.05 and |logFC|*>* 1.0 were employed as the cutoff criteria representing a significant difference. Common DEGs between the datasets were found via a Venn diagram (http://bioinformatics.psb.ugent.be/webtools/Venn/). We used the STRING (http://string-db.org/) online tool to assess DEG-encoded proteins and protein–protein interaction (PPI) networks. Cytoscape Version 3.7.2 (10.1021/acs.jproteome.8b00702) was used to visualize the PPI networks. To identify the hub genes, we used the CytoHubba plug-in Version 0.1 to explore significant nodes in the PPI networks. The cBioPortal for Cancer Genomics (https://www.cbioportal.org/) online tool was used to present information on the genetic alteration of hub genes.

### Perturbation effects of the hub genes on PDAC cell lines

The Cancer Dependency Map (DepMap) database (https://depmap.org/portal/) was used to explore the perturbation effects of the ten hub genes on 44 different PDAC cell lines. The CERES dependency score, which is based on data from a cell depletion assay, was used to evaluate the effect. A low CERES score indicates a higher likelihood that the gene of interest is essential in a given cell line. A score of 0 indicates that a gene is not essential, and a score of -1 is comparable to the median of all pan-essential genes.

### Survival analysis and tissue expression analysis of NUSAP1

Gene Expression Profiling Interactive Analysis (GEPIA) (http://gepia.cancer-pku.cn/) was utilized to show the expression of NUSAP1 in PDAC tumor tissue and normal tissue. Furthermore, survival analysis was performed via GEPIA using data from the TCGA and Genotype-Tissue Expression (GTEx) (https://commonfund.nih.gov/gtex) databases.

### Associations between NUSAP1 expression and genome heterogeneity

To identify the regulatory role of NUSAP1 expression in PDAC, we integrated NUSAP1 gene expression data in the TCGA with data for other variables. Tumor mutation burden (TMB), microsatellite instability (MSI), homologous recombination deficiency (HRD), and neoantigen were used to evaluate the relationship between NUSAP1 expression, tumor mutation load and treatment sensitivity [12]. Mutant-allele tumor heterogeneity (MATH), purity, ploidy, and loss of heterozygosity (LOH) were used to assess the association between NUSAP1 expression and tumor heterogeneity [13].

### Correlation of immune cell infiltration and NUSAP1 expression

Immune cell infiltration in PDAC was estimated from RNA-sequencing data using CIBERSORT (https://cibersortx.stanford.edu/), a versatile computational method for quantifying cell fractions from bulk tissue gene expression profiles (GEPs). A violin plot was drawn in R software Version 4.2.0 (R Foundation for Statistical Computing, Vienna, Austria; https://www.r-project.org/) [14].

### DEG analysis

Analysis of DEGs between the high NUSAP1 expression group and the low NUSAP1 expression group of PDAC samples in the TCGA database was performed in R software, and an adjusted *p* value of < 0.05 and |logFC|> 2.0 were employed as the cutoff criteria. Then, Gene Ontology (GO) and Kyoto Encyclopedia of Genes and Genomes (KEGG) enrichment analyses of these DEGs were performed by Metascape (http://metascape.org).

### PDAC tissue microarray (TMA) and immunohistochemistry (IHC)

TMAs were generated from samples from PDAC patients who were clearly diagnosed through surgical pathology at the Fudan University Shanghai Cancer Center (FUSCC) between February and September 2017. Patients who received medical treatment (e.g., radiotherapy or chemotherapy) before sampling or had a coexisting secondary tumor were excluded. The corresponding clinical data of the patients, including age, sex, tumor location, tumor size, lymph node status, pathologic diagnosis, TNM stage, survival time and outcomes, were recorded. All procedures were performed after obtaining approval from the Clinical Research Ethics Committee of FUSCC, and informed consent was obtained from each patient prior to the analyses. Two independent pathologists conducted the strict pathological diagnoses and postoperative follow-ups. IHC staining with antibodies against NUSAP1 was performed to detect protein expression levels using standard procedures. Protein expression levels were calculated by multiplying the positivity (0, < 5% of the total cells; 1, 5–25%; 2, 25–50%; 3, 50–75%; and 4, > 75%) and intensity scores (0, no coloration; 1, pale yellow; 2, yellow; and 3, clay bank) and were classified as follows: negative (0, −); weakly positive (1–3, +); moderately positive (4–6, ++); and strongly positive (> 6, +++). Then, we divided the patients into two groups (−/+, low expression and ++/+++, high expression) and performed survival analyses [15].

### Cell lines and cell culture

The PANC-1 and CAPAN-1 cell lines were purchased from The Cell Bank of Type Culture Collection of the Chinese Academy of Sciences. PANC-1 cells were maintained at 37 °C with 5% CO_2_ and cultured in DMEM supplemented with 10% FBS (both Gibco; Thermo Fisher Scientific, Inc.). CAPAN-1 cells were maintained at 37 °C with 5% CO2 and cultured in IMDM supplemented with 10% FBS (both Gibco; Thermo Fisher Scientific, Inc.).

### Transfection

The siRNA targeting NUSAP1 and scrambled negative control siRNA (si-NC) were purchased from Santa Cruz Biotechnology, Inc. (cat. nos. sc-93,396 and sc-37,007). Transfection was performed using standard protocols for Lipofectamine® 3000 (Invitrogen; Thermo Fisher Scientific, Inc.). Lipofectamine 3000 reagent and siRNAs were diluted separately with OPTI-MEM (Gibco; Thermo Fisher Scientific, Inc.) in a centrifuge tube, and then Lipofectamine 3000 and siRNAs were mixed and incubated for 15 min at room temperature. Subsequently, the complex was added to the cells and incubated for 48 h at 37 °C [16]. Lentiviral vectors containing NUSAP1 shRNA, NC shRNA and NUSAP1 OE plasmids were obtained from HanBio (China) and transfected into cells according to the manufacturer’s instructions. Stable cells transfected with lentivirus were selected with puromycin (2 µg/mL).

### Quantitative real-time PCR

Quantitative real-time PCR was performed as described previously [16]. All reactions were run in triplicate. RNA was extracted from the cell line preserved in RNAlater using the SteadyPure Universal RNA Extraction Kit (AG21017).

### Western blot analysis

Western blotting was performed as described in our previous study [17, 18]. The antibodies used in the present study were against NUSAP1 (1:3000; Proteintech), GAPDH (1:5000; Abcam), E-cadherin (1:1000; CST), ZEB1 (1:1000; CST), Claudin 1 (1:1000; CST), Snail (1:1000; CST), Zo 1 (1:1000; CST), AMPKα (1:1000; CST) and Phospho-AMPKα (1:1000; CST). The membranes of western blotting were cut to a suitable size prior to binding with antibodies.

### CCK-8 assay

The cells were seeded into 96-well plates at the logarithmic growth stage. After 24 h, 10 µL CCK-8 (Beyotime, Shanghai, China) solution was added to each well, and the cells were further cultured for 2 h. The absorbance value at 450 nm was detected under a microplate reader.

### EdU assay

An EdU incorporation assay was conducted using the BeyoClick EdU Cell Proliferation Kit (cat #C0078S; Beyotime Biotechnology) following the instructions. Briefly, the cells were cultured with 10 µM EdU for 4 h at 37 °C with 5% CO_2_. The cells were then fixed and permeabilized. After washing with PBS three times, the cells were incubated with Click Additive Solution for 30 min at room temperature. Finally, fluorescent images were obtained by a confocal laser scanning microscope.

### Colony formation assays

For colony formation assays, 1000 cells were seeded in 6-well plates. After 14 days, colonies were fixed with 4% paraformaldehyde and stained with crystal violet staining solution (cat #C0121; Beyotime Biotechnology, Shanghai, China). Images and colony counts were obtained using a colony counting machine (Gel Count; Oxford Optronix, UK) [15].

### Flow cytometry

Cells were stained by using a FITC Annexin V Apoptosis Detection Kit (BD, La Jolla, CA, USA) complied with the manufacturer’s instructions and counted using a FACSCalibur flow cytometer to detect apoptotic rate.

### Cell migration and invasion assay

Wound healing assays was used to assess the migration ability of PDAC cells. The cell line was seeded in six-well plates and scratched with a sterile pipette tip, and then the cells were washed with PBS. DMEM or IMDM containing 2% FBS was added to each well. The images were sequentially captured at 0, 12, and 24 h of cultivation.

The Transwell assay was used to assess the migration and the invasion ability of PDAC cells. For migration assays, 6 × 10^4^ cells suspended in 200 µL serum-free medium were added to the upper chamber of the Transwell chamber, each of which included a Tewksbury multiporous polycarbonate membrane (8-mm pore size) insert, and medium containing 10% FBS as a chemical attractant was placed in the bottom chamber. According to the manufacturer’s instructions, cell invasion was detected using a Transwell chamber coated with Matrigel (1:100 in DMEM; BD Biosciences, USA). A total of 2 × 10^5^ cells in serum-free medium were added to the upper chamber, and the lower chamber contained 500 µL of 20% FBS-supplemented medium. After culturing the cells for 24 h, they were fixed with 4% paraformaldehyde for 30 min. The migrated cells were stained with crystal violet for 20 min and washed with PBS three times.

### Animal studies

Four- to five-week-old female nude mice were obtained from Shanghai SLAC Laboratory (Shanghai, China). Ten mice were randomly divided into two groups (5 mice/group): the NC group and the KD group, approximately 5 × 10^6^ cells in 200ul PBS were subcutaneously inoculated on right flank of the mice. Following the formation of palpable tumors, we tested the tumor size every 4 days and calculated the tumor volume following the formula: length × width^2^ × 0.5. At 4 weeks post implantation, the tumor specimens were surgically dissected, fixed with paraformaldehyde and then subjected to immunohistochemical staining. NUSAP1, Ki-67, BAX, and Cleaved caspase-3 were evaluated, and the calculation methods for protein expression levels were the same as those used for TMAs. The protocol was approved by the Committee on the Ethics of Animal Experiments of Fudan University, and the study is reported in accordance with ARRIVE guidelines.

### Statistics

Statistical analyses were conducted using SPSS Statistics Version 25.0.0 (IBM Inc., Chicago, IL, USA; https://www.ibm.com/docs/en/spss-statistics/25.0.0). Statistical significance was determined by Student’s t test, chi-square test, log-rank test, and Pearson correlation analysis. Differences with *P* < 0.05 were considered to be statistically significant.

## Results

### Identification of hub genes in PDAC tumorigenesis

**Fig. 1:**
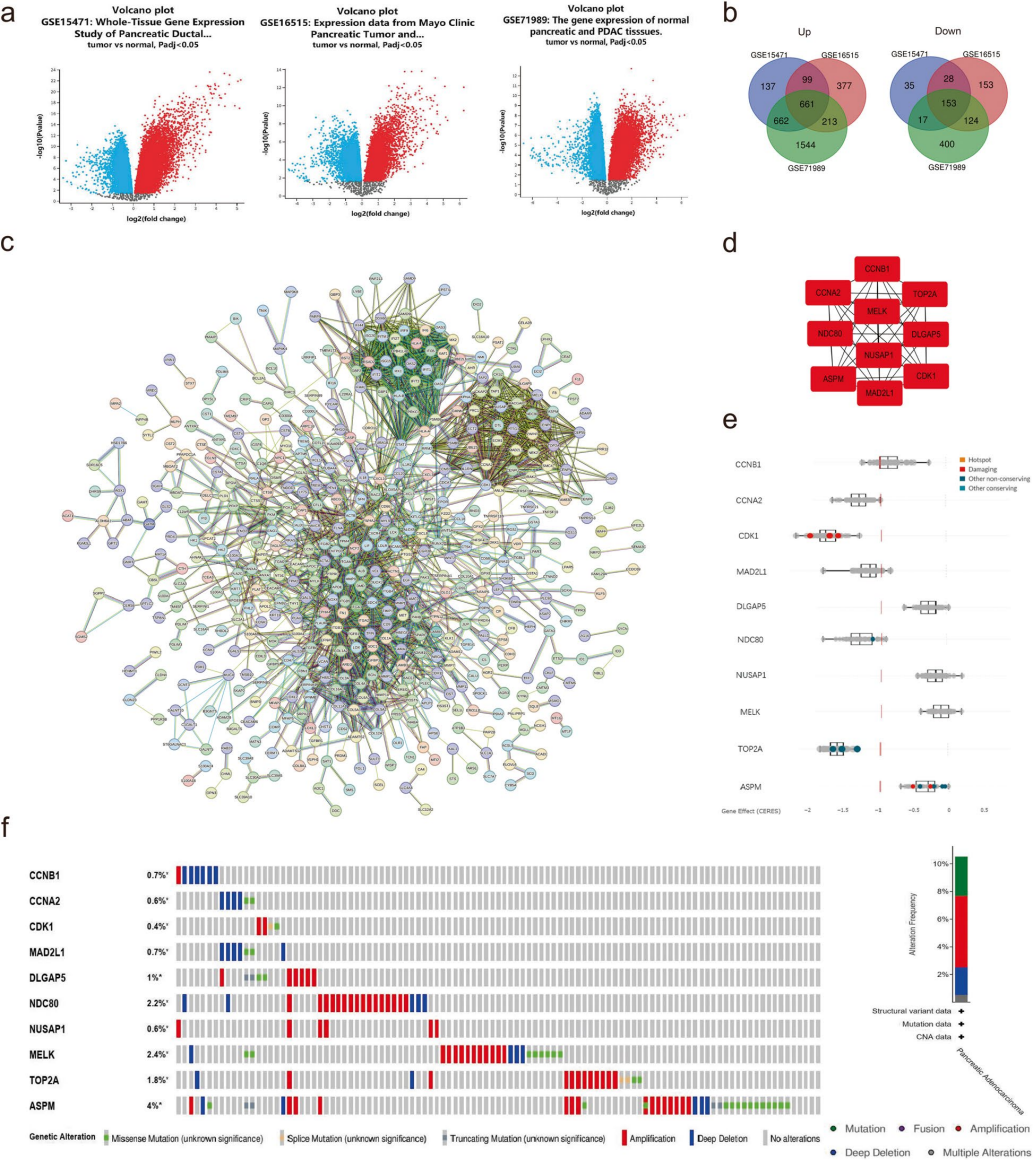
NUSAP1 was among the top 10 key genes in PDAC. **a** Volcano plots of DEGs in each GEO dataset were drawn using GEO2R. Red represents the genes that were significantly upregulated, and blue represents the downregulated genes in PDAC samples. Black dots represent the genes that were not significantly upregulated or downregulated in PDAC samples. **b** The Venn diagram shows the number of common upregulated and downregulated DEGs shared by the three GEO datasets. **c** STRING PPI network of common DEGs identified from three GEO datasets. **d** Subnetwork of the top 10 hub genes from the PPI network using Cytoscape software. **e** Perturbation effects of the hub genes on 44 PDAC cell lines. **f** Information on the genetic alterations of the hub genes

Three expression profiles (GSE15471, GSE16515 and GSE71989) were obtained from the GEO database. The details of the above datasets are presented in Table 1. The volcano plot shows the DEGs between PDAC tissues and normal tissues (Fig. 1a). The Venn diagram of the results from all three datasets indicated a total of 814 common DEGs, which consisted of 661 upregulated genes and 153 downregulated genes (Fig. 1b). Using the STRING application and Cytoscape software, the top 10 hub genes ranked by the degree of connectivity with other proteins were selected (Fig. 1c and d). All ten hub genes had negative median CERES scores for the 44 PDAC cell lines, which means that the ten genes may fuel PDAC proliferation (Fig. 1e). The cBioPortal for Cancer Genomics was used to assess the genetic alteration of the 10 hub genes. As presented in Fig. 1f, the hub genes were altered in 14.4% of the samples. These alterations included amplification, deep deletion, missense mutation, splice mutation, and truncating mutation. Among the different types of alterations, amplification accounted for the highest percentage (Fig. 1f). We searched PUBMED for studies on the roles of the top 10 hub genes. Some of them (CDK1 [19], TOP2A [20], CCNA2 [21], ASPM [22], CCNB1 [23]) have been studied to varying degrees concerning their relationship with PDAC. Considering the background and potential functions of the remaining genes, we selected NUSAP1 for further validation and exploration.

**Table 1:** Detailed information on the GEO microarray profiles of PDAC patients

Profile no.	Type	Source	Cases	Controls	Platform	Annotation platform
GSE15471	RNA	PDAC	36	36	GPL570	Affymetrix Human Genome U133 Plus 2.0 Array
GSE16515	RNA	PDAC	36	16	GPL570	Affymetrix Human Genome U133 Plus 2.0 Array
GSE71989	RNA	PDAC	14	8	GPL570	Affymetrix Human Genome U133 Plus 2.0 Array

### Correlation between NUSAP1 expression and genome heterogeneity in PDAC

**Fig. 2:**
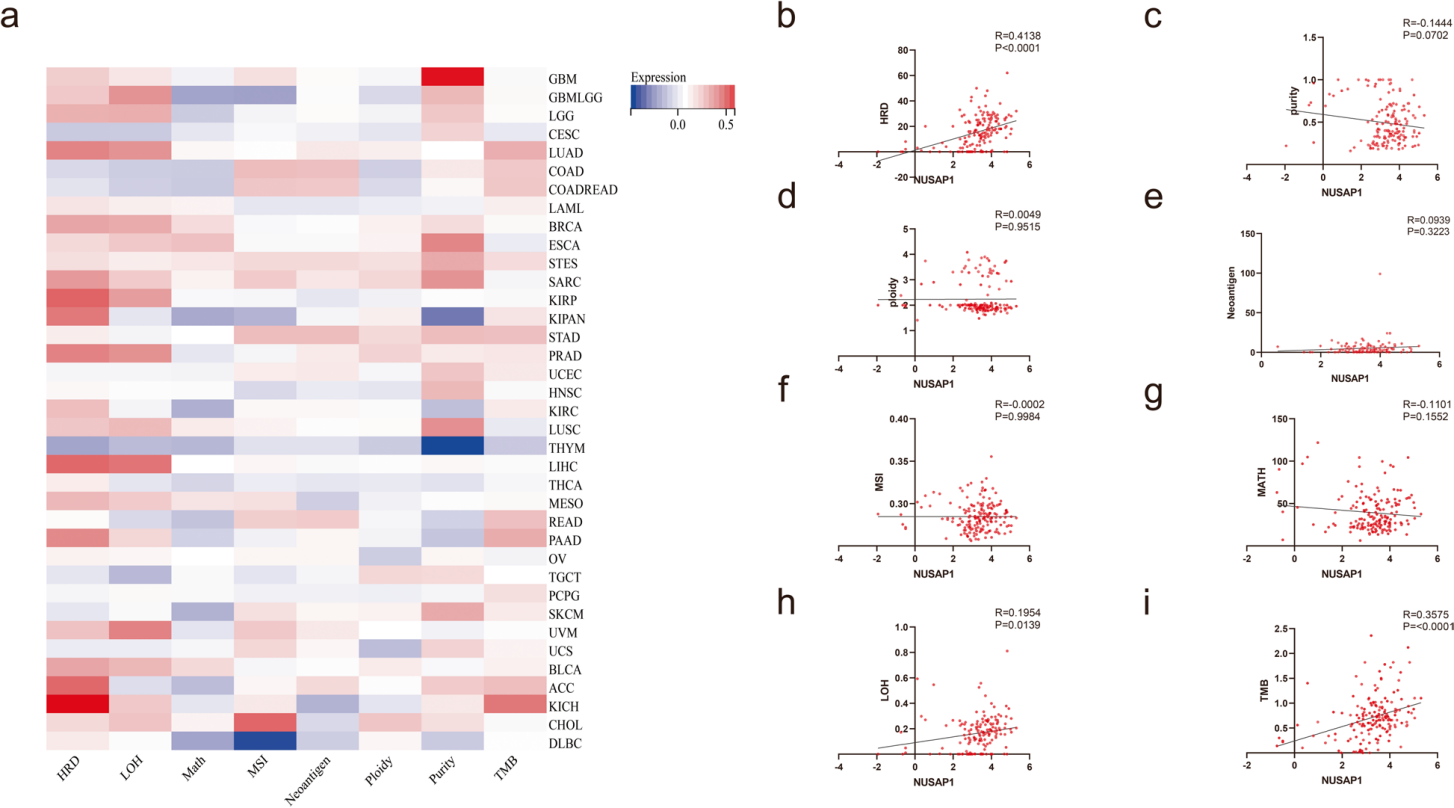
Correlations between NUSAP1 expression and genome heterogeneity in PDAC. **a** Pan -cancer analysis of the correlation between NUSAP1 and tumor genome heterogeneity. **b** Homologous recombination deficiency (HRD). **c** Purity. **d** Ploidy. **e** Neoantigen load. **f** Microsatellite instability (MSI). **g** Mutant-allele tumor heterogeneity (MATH). **h** Loss of heterozygosity (LOH). **i** Tumor mutation burden (TMB). Pearson correlation analysis was used to analyze correlations and obtain P and R values

Genome heterogeneity is important for PDAC progression and drug resistance. We performed pancancer analysis to assess the correlation between NUSAP1 expression and genome heterogeneity markers (Fig. 2a). As shown in Fig. 2b-i, NUSAP1 was positively related to tumor mutation burden (TMB) (*R* = 0.3575, *P* < 0.0001), loss of heterozygosity (LOH) (*R* = 0.1954, *P* = 0.0139) and homologous recombination deficiency (HRD) (*R* = 0.4138, *P* < 0.0001) in PDAC. However, NUSAP1 expression was not significantly related to MATH, neoantigen load, MSI, ploidy or purity.

### Associations between NUSAP1 and the immune microenvironment in PDAC

**Fig. 3:**
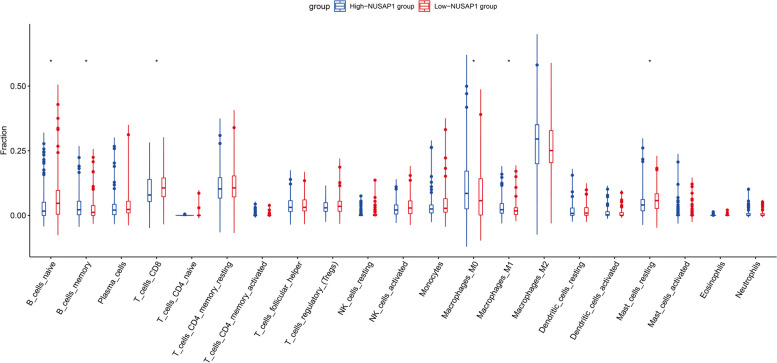
Correlations between the expression of NUSAP1 and the infiltration levels of immune cells in PDAC. Student’s t test was used for comparisons between groups. *P < 0.05

CIBERSORT is an excellent tool for estimating immune cell infiltration and was adopted to evaluate the relative proportion of immune cells in PDAC specimens. As shown in Fig. 3, the highly infiltrating immune cells included naive B cells, M0 macrophages and M2 macrophages. Among the 21 types of immune cells, the relative proportions of memory B cells, M0 macrophages and M1 macrophages had significant positive correlations with NUSAP1 expression (*P* < 0.05, respectively), while the relative proportions of naive B cells, CD8 T cells and resting mast cells had significant negative correlations (*P* < 0.05, respectively).

### Detection of differentially expressed genes and enrichment analysis based on TCGA-PAAD cohort

**Fig. 4:**
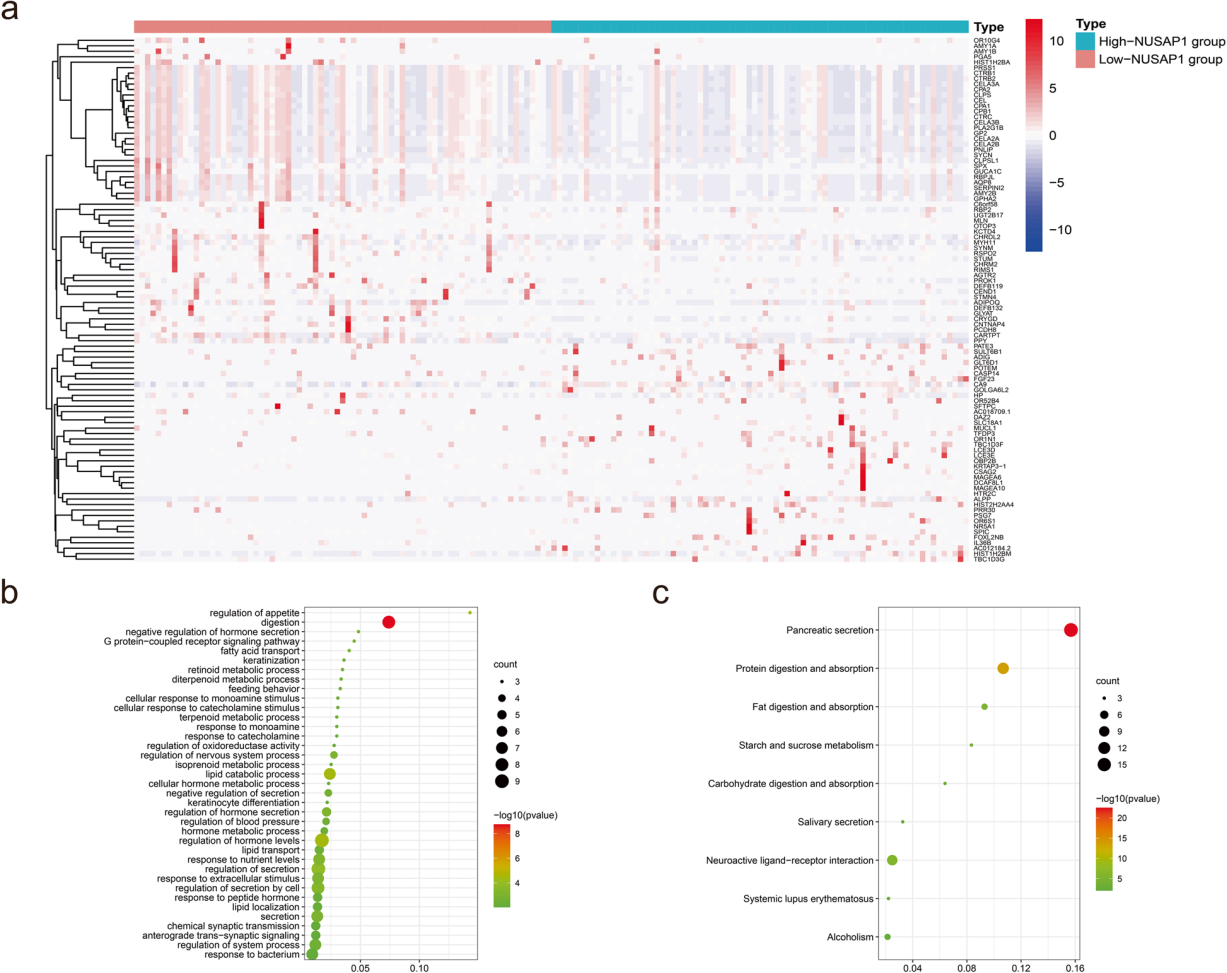
DEGs between PDAC samples with high and low NUSAP1 expression in the TCGA database. **a** Heatmap of the 96 DEGs between the two groups in the TCGA. **b** GO enrichment results for the 96 DEGs. **c** KEGG pathway enrichment results for the 96 DEGs.

Ninety-six DEGs were found between the high NUSAP1 expression group and the low NUSAP1 expression group of PDAC samples from the TCGA dataset (Fig. 4a). Then, GO and KEGG pathway analyses of these DEGs were performed via Metascape. The GO enrichment analysis indicated that the DEGs were mainly enriched in regulation of appetite, digestion, negative regulation of hormone secretion, G protein-coupled receptor signaling pathway and fatty acid transport. The KEGG pathway enrichment analysis indicated that the DEGs were mainly enriched in pancreatic secretion, protein digestion and absorption, and fat digestion and absorption (Fig. 4b and c).

### NUSAP1 is overexpressed in PDAC tissues and predicts a poor prognosis in PDAC patients

**Fig. 5:**
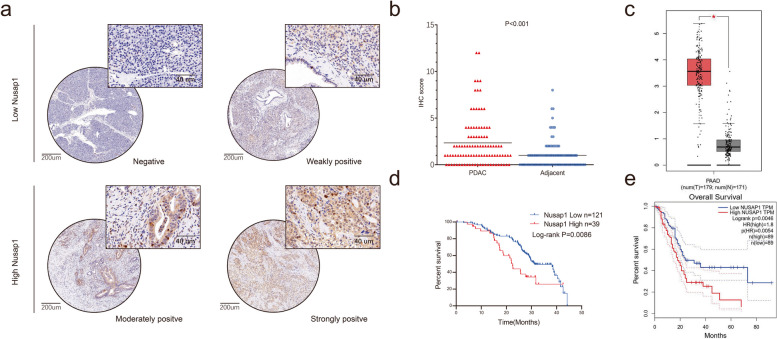
NUSAP1 expression is increased in PDAC tissues. **a** Representative images of IHC staining for NUSAP1 in TMAs (scale bar, 200 μm; inset scale bar, 40 μm). **b** NUSAP1 expression in PDAC and adjacent normal tissues, as determined by the IHC score (*n* = 102, Student’s t test, *P* < 0.001). **c** Box plot analyses comparing the expression levels of NUSAP1 in patient PDAC tissues (red, *n* = 179) and normal tissues (gray, *n* = 171) in the GEPIA database (Student’s t test, *P* < 0.05). **d** The OS of patients with PDAC from our center was assessed using Kaplan–Meier analysis based on NUSAP1 expression (*n* = 160, log-rank test, *P* = 0.0086). (e) OS analyses of PDAC patients according to the expression of NUSAP1 (high versus low) in the GEPIA database (*n* = 178, log-rank test, *P* = 0.0046)

NUSAP1 expression was evaluated through TMAs generated from samples from 160 PDAC patients (including 102 pairs of samples) to further validate the IHC results (Fig. 5a; Table 2). According to the IHC score, NUSAP1 was overexpressed in the tumor tissues (t = 4.473, *P* < 0.001) (Fig. 5b). An analysis of the clinical characteristics of PDAC revealed that high NUSAP1 expression was related to high tumor–node–metastasis (TNM) stage (χ^2^ = 8.971, *P* = 0.003) (Table 3). Furthermore, Kaplan–Meier survival curves revealed an obvious correlation between low NUSAP1 expression and a better prognosis in patients with PDAC (log-rank χ^2^ = 6.899, *P* = 0.0086) (Fig. 5d). Using the GEPIA database, we found that NUSAP1 was highly expressed in tumor tissues compared with normal tissues (*P* < 0.05) (Fig. 5c), and a high expression level of NUSAP1 was correlated with an unfavorable prognosis in PDAC patients (*P* = 0.0046) (Fig. 5e). The GEPIA results were consistent with the TMA results. All these results indicated that NUSAP1 is an oncogenic protein involved in PDAC tumorigenesis.

**Table 2:** Summary of the NUSAP1 expression levels in TMAs

	NUSAP1 expression				Total
	Low		High		
	-	+	++	+++	
PDAC/Normal	40/25	81/53	22/16	17/8	
Total	121/78		39/24		160/102

**Table 3:** Clinicopathological features of PDAC patients and correlations with NUSAP1 expression

Characteristics	No.	Low NUSAP1	High NUSAP1	*P* value
		score (-/+) (*n* = 121)	score (++/+++) (*n* = 39)	
Age				
< 60 years	59	47	12	0.363
≥ 60 years	101	74	27	
Sex				
Female	68	52	16	0.830
Male	92	69	23	
Tumor location				
Head	89	69	20	0.530
Body and tail	71	52	19	
Tumor size				
<4.0 cm	103	77	26	0.731
≥ 4.0 cm	57	44	13	
Lymph node status				
Negative	80	58	22	0.357
Positive	80	63	17	
TNM stage				
I and II	135	108	27	0.003
III	25	13	12	

### NUSAP1 promotes the proliferation, migration and invasion of PDAC cells

**Fig. 6:**
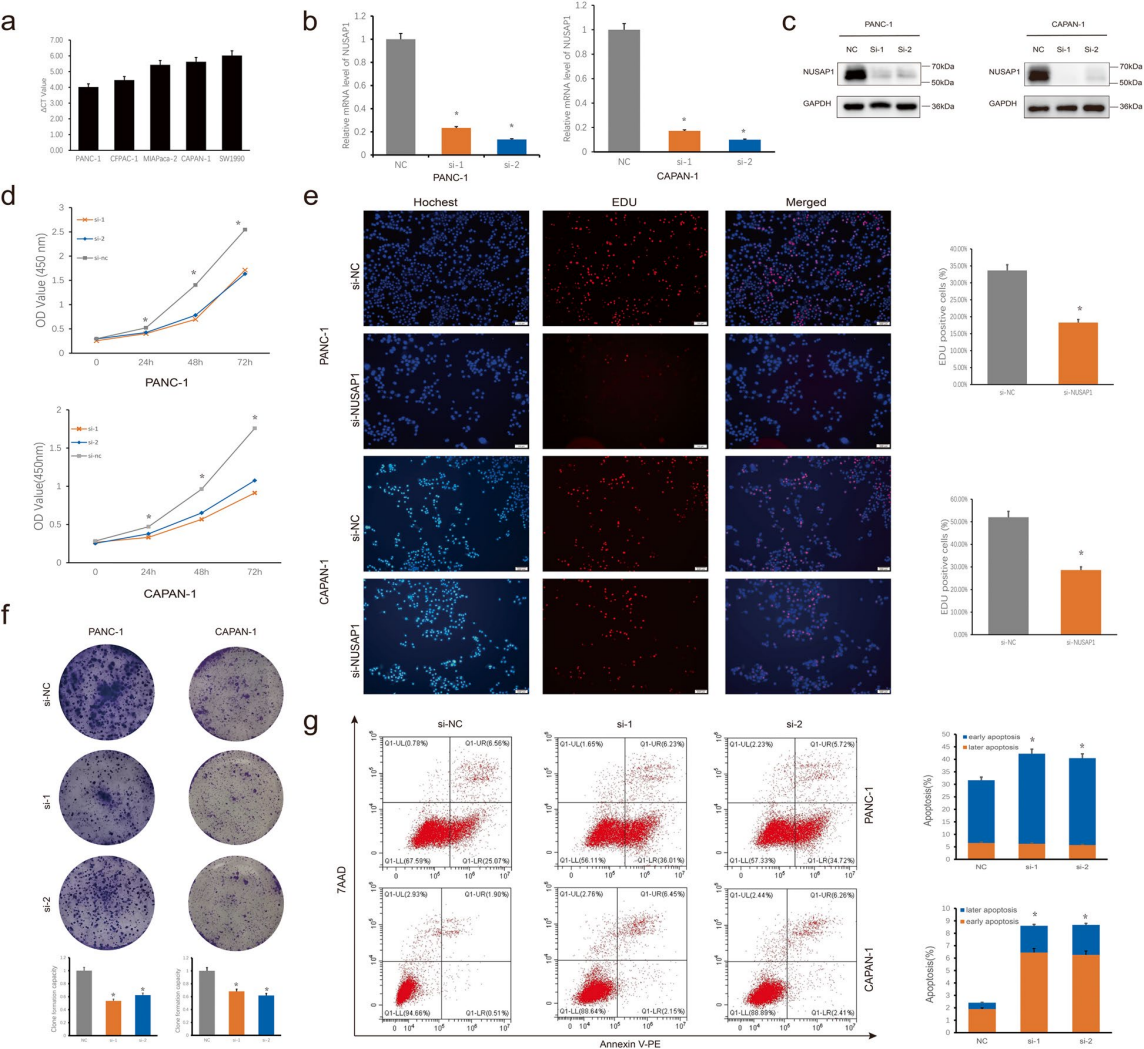
Decreasing NUSAP1 expression inhibits PDAC cell proliferation. **a** Relative expression levels of NUSAP1 among the PDAC cell lines. **b** mRNA expression levels of NUSAP1 in PANC-1 and CAPAN-1 cells were detected by reverse transcription quantitative PCR after transfection of si-NUSAP1. **c** The protein expression level of NUSAP1 decreased significantly after transfection of cells with si-NUSAP1. **d** The proliferation of PDAC cells was measured using CCK-8 assays. **e** Visualization of DNA replication by EdU incorporation. Cell nuclei stained red represent DNA replication. **f** A significant reduction in colony formation ability was observed after transfection with si-NUSAP1. **g** Apoptosis rates of the si-NUSAP1 cell lines. Student’s t test was used for comparisons between groups. All experiments were performed in triplicate. *P < 0.05. NC, negative control; si, small interfering RNA.

**Fig. 7:**
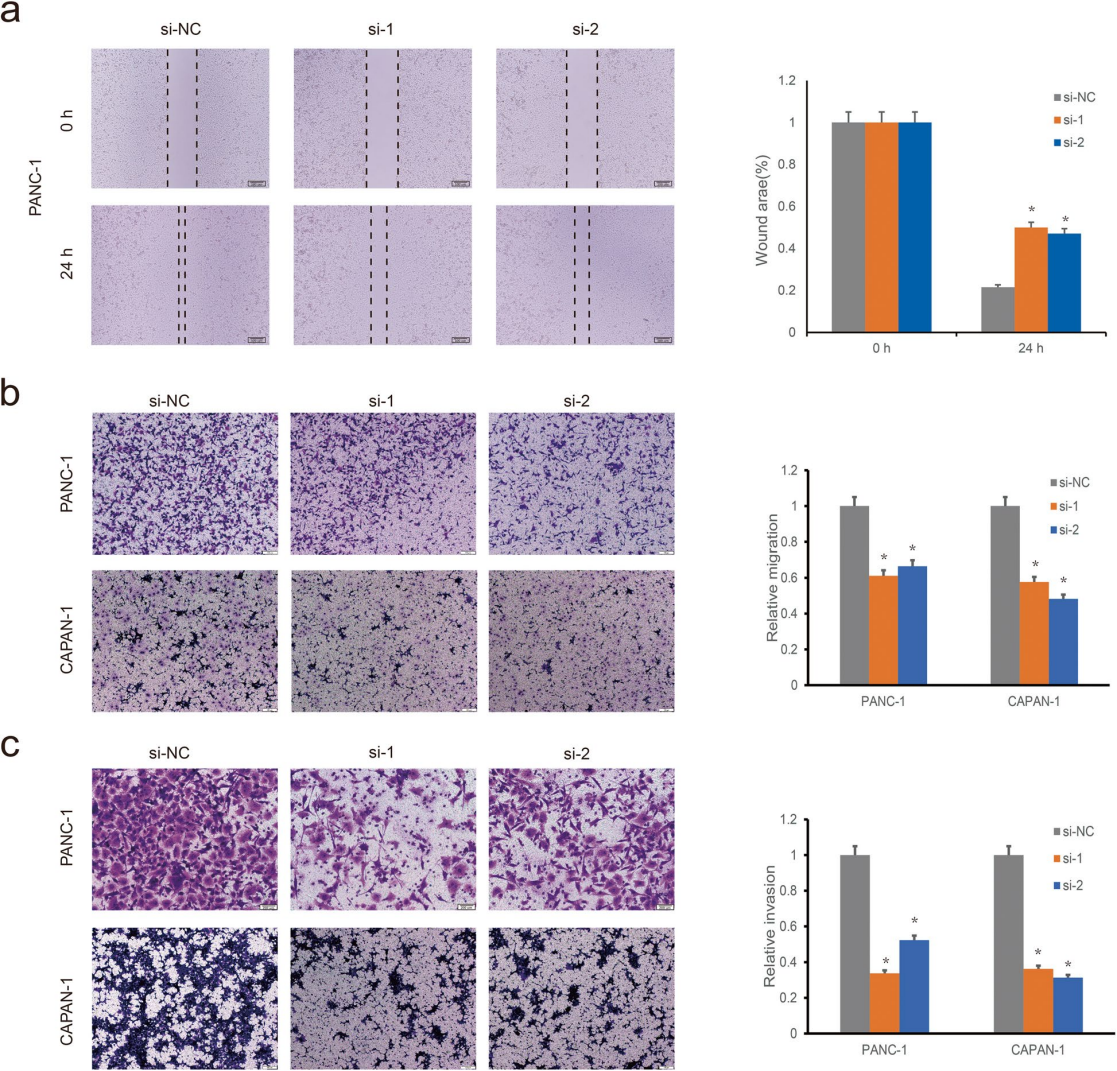
Decreasing the expression of NUSAP1 inhibits the migration and invasion of PDAC cells. **a** Migration of PANC-1 cells in the si-NC and si-NUSAP1 groups according to Wound healing assays. **b** Migration of PANC-1 and CAPAN-1 cells in the si-NC and si-NUSAP1 groups according to Transwell assays. **c** Invasion of PANC-1 and CAPAN-1 cells in the si-NC and si-NUSAP1 groups according to Transwell assays. Student’s t test was used for comparisons between groups. All experiments were performed in triplicate. *P < 0.05. NC, negative control; si, small interfering RNA.

We assessed the expression levels of NUSAP1 in five PDAC cell lines by quantitative real-time PCR and found that the PANC-1 cell line exhibited the highest NUSAP1 expression (Fig. 6a). Among the 5 PDAC cell lines, besides PANC-1, CAPAN-1 was also chosen for follow-up experiments because it showed the lowest CERES score for NUSAP1 in DepMap. As shown in Fig. 6b and c, si-NUSAP1 reduced NUSAP1 expression in both PANC-1 and CAPAN-1 cells. Cell proliferation was assessed via CCK-8 assays, colony formation assays and EdU incorporation assays. As shown in Fig. 6d, the proliferation of PANC-1 and CAPAN-1 cells was suppressed in the si-NUSAP1 group compared with the si-NC group in CCK-8 assays. Similarly, colony formation and EdU incorporation assays revealed the attenuation of proliferation and DNA replication in the si-NUSAP1 groups (Fig. 6e and f). Furthermore, apoptosis assays revealed NUSAP1 knockdown increased the apoptosis rate of PDAC cells (Fig. 6g). We evaluated the impact of NUSAP1 on the migration and invasion of PANC-1 and CAPAN-1 cells by Wound healing and Transwell assays. The results of Wound healing and Transwell assays showed that the migration and invasion abilities of these two cell lines decreased after NUSAP1 knockdown, and the differences between NC and si-NUSAP1 cell lines were all significant (Fig. 7).

**Fig. 8:**
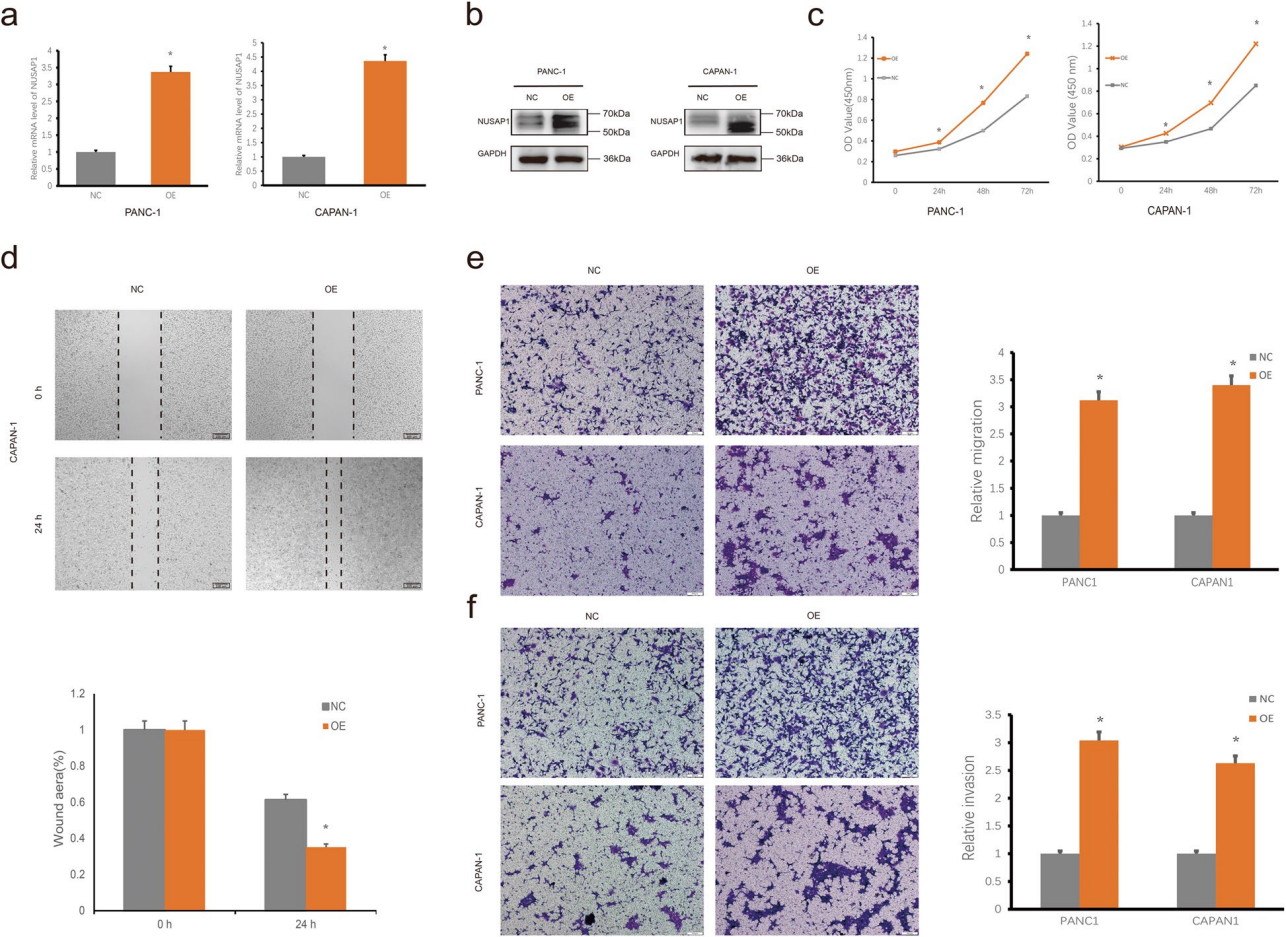
NUSAP1 overexpression promotes the proliferation, migration and invasion of PDAC cells. **a** mRNA expression levels of NUSAP1 in PANC-1 and CAPAN-1 cells were detected by reverse transcription quantitative PCR after transfection of lentiviral vectors containing NUSAP1 OE plasmids. **b** The protein expression level of NUSAP1 increased significantly after transfection with OE-NUSAP1. **c** The proliferation of PDAC cells was measured using CCK-8 assays. **d** Cell migration of CAPAN-1 cells in the NC and OE groups according to Wound healing assays. **e** Cell migration of PANC-1 and CAPAN-1 cells in the NC and OE groups according to Transwell assays. **f** Cell invasion of PANC-1 and CAPAN-1 cells in the NC and OE groups according to Transwell assays. Student’s t test was used for comparisons between groups. All experiments were performed in triplicate. *P < 0.05. NC, negative control; OE, overexpression

In contrast, we found that overexpression of NUSAP1 can promote the proliferation, migration and invasion of PANC-1 and CAPAN-1 cell lines (Fig. 8). These results indicated that NUSAP1 is essential for PDAC tumorigenesis and progression.

### Knockdown of NUSAP1 inhibited PDAC growth in vivo

**Fig. 9:**
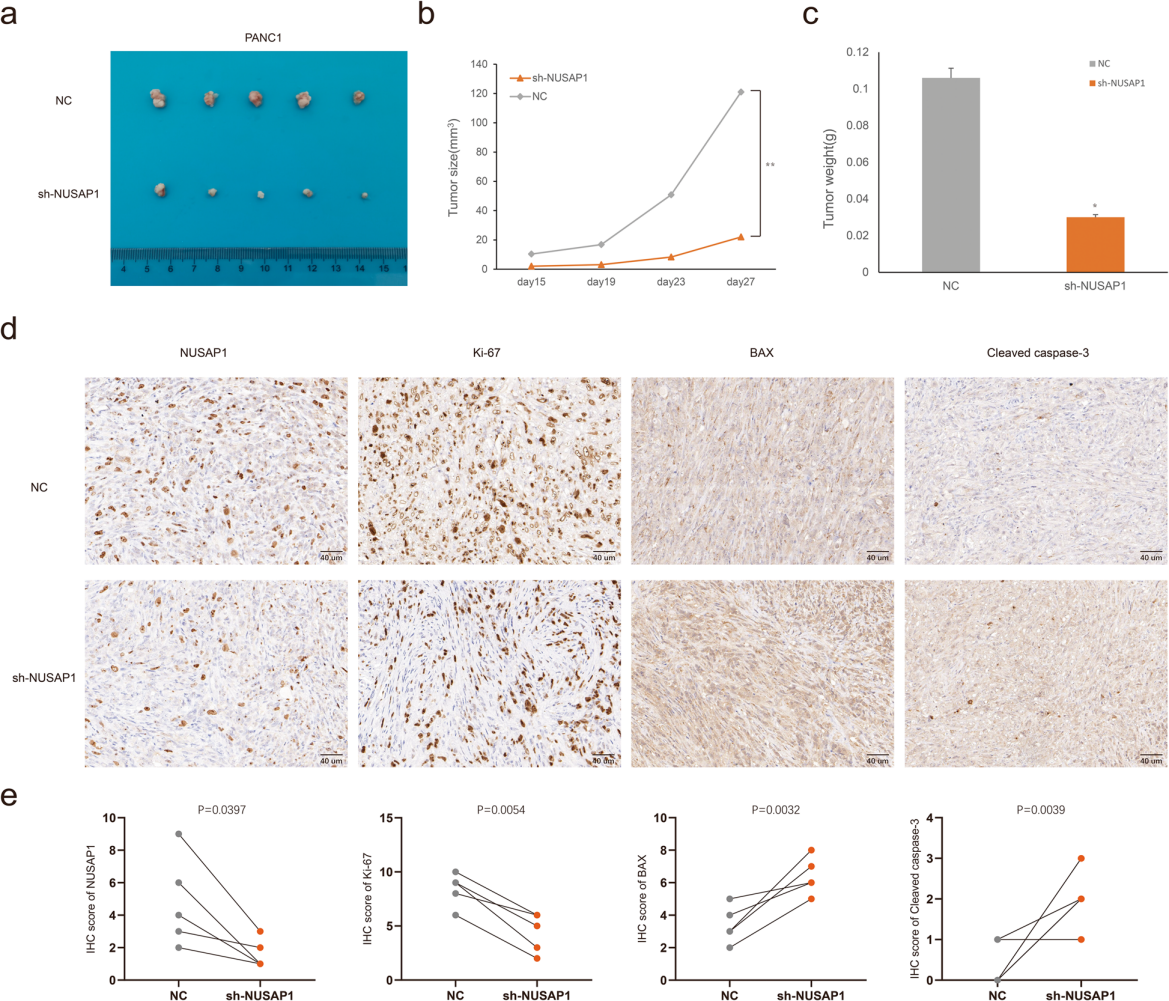
NUSAP1 promoted progression of PDAC in vivo. **a** We randomly divided the mice into the NC and the KD groups (*n* = 5), PANC-1 cells stably transfected with an empty vector or the NUSAP1 shRNA were subcutaneously inoculated in them respectively. Then we treated the mice as described in the Methods. **b** The tumor sizes were tested using Vernier calipers. Tumor growth curves were constructed based on the tumor volumes measured in the mice. **c** Quantification of the average weights of collected tumors from the above experiments. **d** The expression of NUSAP1, Ki-67, BAX and Cleaved caspase-3 were determined in tumor tissue sections from the xenografts using IHC (scale bar, 40 μm, *n* = 5). **e** The expression of NUSAP1, Ki-67, BAX, and Cleaved caspase-3 in tumor tissue between the NC and the KD groups, as determined by the IHC score. Student’s t test was used for comparisons between groups. *P < 0.05, **P < 0.01. NC, negative control; KD, knockdown

In order to further confirm the biological functions of NUSAP1, we established a xenograft tumor model by inoculating PANC-1 cells that transfected with empty vector or the NUSAP1 shRNA into nude mice and monitored tumor size for 28 days. As illustrated in Fig. 9a-c, the tumor growth rate and size were significantly decreased in the sh-NUSAP1 PANC-1 cell groups than in the NC group. Subsequent IHC using antibodies against NUSAP1, Ki-67, BAX and Cleaved caspase-3 showed that NUSAP1 knockdown obviously reduced Ki-67 staining and increased BAX and Cleaved caspase-3 staining in the sh-NUSAP1 group than in the NC group (Ki-67: t = 3.780, *P* = 0.0054; BAX: t = 4.160, *P* = 0.0032; Cleaved caspase-3: t = 4.000, *P* = 0.0039) (Fig. 9d and e).

### NUSAP1 drives the epithelial-mesenchymal transition and reduces AMPK phosphorylation

**Fig. 10:**
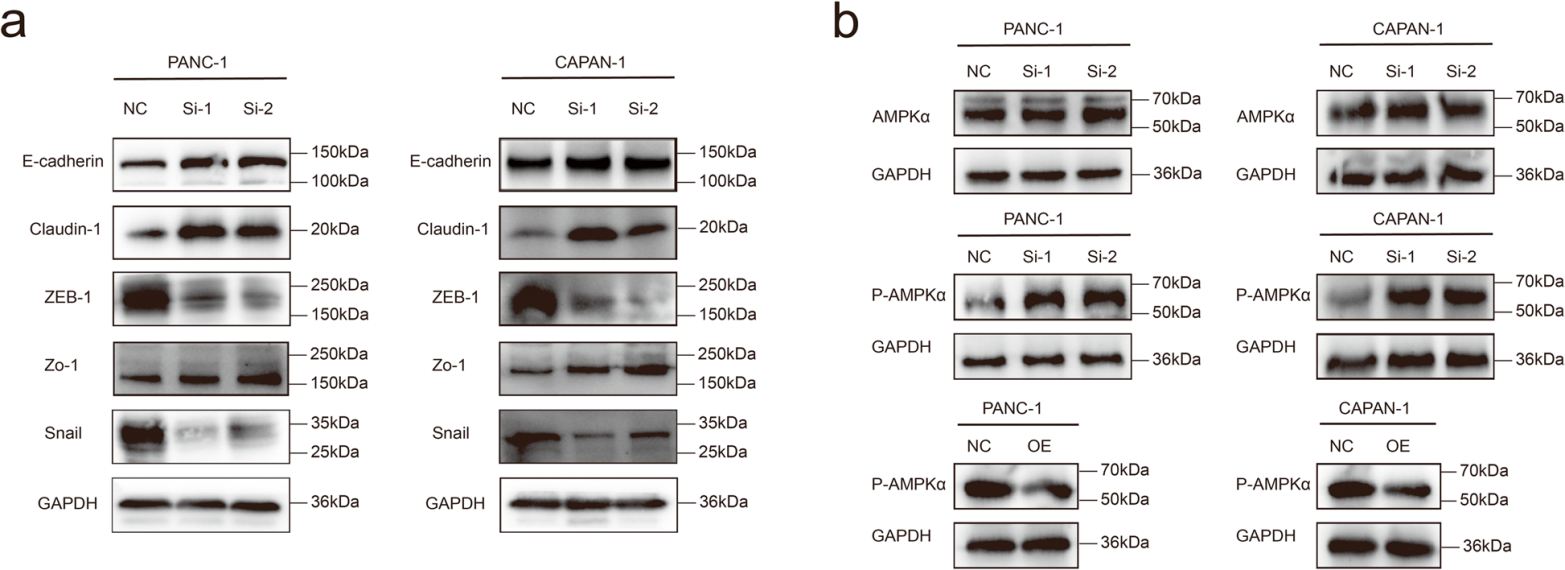
NUSAP1 drives the EMT and reduces AMPK phosphorylation. **a** The expression of epithelial and mesenchymal cell phenotype markers was determined by western blot analysis. **b** Western blot analysis of AMPKα protein expression in NC and NUSAP1-KD cell lines, as well as phospho-AMPKα protein expression in NC, NUSAP1-KD, and NUSAP1-OE cell lines. NC, negative control; KD, knockdown; OE, overexpression

NUSAP1 knockdown PDAC cells exhibited increased E-cadherin, Claudin 1, and Zo 1 expression, which indicates reversal of the epithelial-mesenchymal transition (EMT) phenotype (Fig. 10a). NUSAP1 affect tumor growth by regulating the AMPK signaling pathway has been reported in tumors such as breast cancer and gastric cancer [9, 24]. In our study, western blotting was used to examine the expression variants of AMPK phosphorylation in NUSAP1-KD or NUSAP1-OE cell lines. Figure 10b shows that P-AMPKα was upregulated in NUSAP1-KD cell lines and downregulated in NUSAP1-OE cell lines.

## Discussion

Although great progress in surgical and medical treatment has been made for PDAC, it still has a dismal prognosis, highlighted by a low survival rate and unfavorable therapeutic efficacy [25]. PDAC-related death is mainly attributed to a lack of early detection methods, the high risk for metastasis, and chemotherapy resistance. Therefore, exploring reliable biomarkers and precise molecular mechanisms for the early diagnosis, treatment, and prognosis evaluation of PDAC is urgent [26]. NUSAP1 was reported to be a potential biomarker for PDAC diagnosis and prognosis evaluation in previous studies [27]. It has been reported to be associated with mitosis, which is an integral cell process that requires great accuracy to ensure correct and stable chromosome replication [28]. NUSAP1 participates in regulating the Wnt/β-catenin signaling pathway and is expressed at higher levels in breast cancer and liver cancer tissues than in corresponding normal tissues [29, 30]. There are only a few reports on the role of NUSAP1 in the occurrence and progression of pancreatic cancer. In our study, we found that NUSAP1 promoted the proliferation, epithelial mesenchymal transition, migration and invasion of cancer cells in vitro. TMA analysis revealed high expression of NUSAP1 in tumor tissues compared with normal tissues, and this upregulation was associated with a poor prognosis. Our results confirmed that NUSAP1 plays a role as an oncogene in PDAC.

Novel methods are being continually established for the identification of the molecular mechanisms of cancer. RNA sequencing and cDNA microarray are both high-throughput screening (HTS) techniques and are widely used to explore the mechanisms involved in carcinogenesis and tumor progression [31]. A large amount of corresponding data from HTS techniques is stored in several public databases, such as the TCGA, International Cancer Genome Consortium (ICGC) (https://dcc.icgc.org/) and GEO. Compared to analysis of individual HTS datasets, integration of multiple HTS datasets (cDNA microarray and RNA sequencing) is considered to increase the reliability of results [32]. In the present study, three GEO datasets, namely, GSE15471, GSE16515 and GSE71989, were selected to screen DEGs. PPI network and module analyses were used to identify the top 10 high-scoring hub genes (CCNB1, CCNA2, CDK1, MAD2L1, DLGAP5, NDC80, NUSAP1, MELK, TOP2A and ASPM), which confirmed that NUSAP1 may play an important role in the development of PDAC. The cBioPortal for Cancer Genomics, an open-access resource for interactive exploration of multidimensional cancer genomics datasets that provides access to data from more than 5,000 tumor samples [33], was used to show mutation information of the hub genes. DepMap is an accessible website based on large-scale multiomics screening projects, including the Cancer Cell Line Encyclopedia (CCLE), the PRISM Repurposing dataset and the Achilles Project [34]. CERES (CRISPR) scores, representing the effect of each gene in a given set, were determined by screening experiments. Simply, the score evaluates the effect size of knocking out or knocking down a gene while normalizing expression against the distribution of pan-essential and nonessential genes. A negative score indicates that the cell line grows slower after experimental manipulation, while a positive score indicates that the cell line grows faster [35]. The tumor immune microenvironment (TIME) has gradually attracted attention, and analysis of the TIME will contribute to the improvement of immunotherapy [36]. Some researchers revealed that the TIME could be used as a main prognostic indicator and could enhance the potential of precision treatments [37]. We also found that NUSAP1 can cause changes in the TIME of PDAC, which may be one of the ways in which this gene plays its role. Bioinformatics analysis could assist with exploring the biomarkers and the mechanisms underlying the tumorigenesis and progression of cancer and may play an increasingly important role in future research [8].

As an energy sensor, AMPK is activated when the intracellular ATP level is insufficient and triggers a series of downstream responses that inhibit rapid cell proliferation and has been frequently identified as a potential target in anticancer treatment [38]. In the context of metabolic regulation, AMPKα is a key molecule responsible for energy regulation. The AMPKα-Sirt1-FGF21 cascade has a strong correlation to metabolic energy modulation. AMPK activation can increase glucose uptake and decrease hyperglycaemia by promoting energy expenditure, together with the increase in insulin sensitivity to attenuate metabolic stresses [39]. Although there is evidence suggesting that AMPK might help cancer cells survive under certain circumstances, there is more support in the literature for the notion that AMPK acts as a tumor suppressor by leading to cell growth inhibition and cell cycle arrest [40]. Song’s study revealed that Hernandezine activates autophagy and induces autophagic cell death in PDAC cells by promoting ROS generation, activating the AMPK signaling pathway and inhibiting the mTOR/p70S6K signaling pathway [41]. Notably, activation of AMPK is dependent on the phosphorylation of AMPKα at Thr-172. Our study firstly found that NUSAP1 affects the proliferation and metastasis of PDAC through inhibition AMPK signaling pathway, which provides a new idea for targeting the NUSAP1/AMPK axis in PDAC research and treatment.

Taken together, the current study demonstrated very interesting and strong evidence that NUSAP1 is a key gene in PDAC and may be an effective novel target for treatment. Therefore, considering the crucial roles of NUSAP1 identified in this study and previous studies mentioned above, further research procedure such as RNA sequencing may be needed and focused on exploring the gene’s precise mechanisms in PDAC for only simple experiments were performed in this study. However, this study provides novel information regarding the role of NUSAP1 in PDAC.

## Conclusions

In summary, NUSAP1 is a hub gene of PDAC. It can promote the growth, migration and invasion of the tumor and is related to a poor prognosis in patients. NUSAP1 drives the epithelial-mesenchymal transition and reduces AMPK phosphorylation. In-depth study of the molecular mechanisms of NUSAP1 in PDAC is needed in the future, and relevant experimental models could be constructed on the basis of these mechanisms.

### Supplementary Information

**Additional file 1: Supplementary Information files. **Original versions of Western blots in Fig. 6c, Fig. 8b, Fig. 10a, and Fig. 10b.

## Data Availability

All data generated or analysed during this study are included in this published article.
